# 5-year results of a newly implemented mechanical circulatory support program for terminal heart failure patients in a Swiss non-cardiac transplant university hospital

**DOI:** 10.1186/s13019-021-01447-5

**Published:** 2021-03-31

**Authors:** Thibault Schaeffer, Otmar Pfister, Constantin Mork, Paul Mohacsi, Florian Rueter, Simon Scheifele, Anne Morgen, Urs Zenklusen, Thomas Doebele, Markus Maurer, Joachim Erb, Jens Fassl, Nadine Cueni, Martin Siegemund, Hans Pargger, Brigitta Gahl, Stefan Osswald, Friedrich Eckstein, Martin Grapow

**Affiliations:** 1 Department of Cardiac Surgery, University Hospital of Basel, Basel, Switzerland; 2 Department of cardiology, University Hospital of Basel, Basel, Switzerland; 3 Department of Anesthesiology, University Hospital of Basel, Basel, Switzerland; 4 Institute of Cardiac Anesthesiology, Heart Center of the Technical University of Dresden, Dresden, Germany; 5 Department of Intensive Care, University Hospital of Basel, Basel, Switzerland; 6 HerzZentrum Hirslanden Zürich, Witellikerstrasse 36, Zürich, Switzerland

**Keywords:** Left ventricular assist device, Destination therapy, Comprehensive heart-failure center

## Abstract

**Background:**

In Switzerland, long-term circulatory support programs have been limited to heart transplant centers. In 2014, to improve the management of patients with end-stage heart failure not eligible for transplantation, we implemented a left ventricular assist device (LVAD) program for destination therapy at the University Hospital of Basel.

**Methods:**

We described the program set-up with practical aspects. Patients aged 65 and above with therapy refractory end-stage heart failure without major contraindication for LVAD implantation were included. Younger patients with bridge-to-candidacy profile were also considered. Using the Kaplan-Meier estimate, we retrospectively analyzed the overall survival and freedom from major adverse events after LVAD implantation. We compared our results to internationally reported data.

**Results:**

Between October 2014 and September 2019, 16 patients received an LVAD in our center. The mean age at implantation was 67.1 years. The mean EuroSCORE II was 24.4% and the median INTERMACS level was 4. Thirteen patients received an LVAD as destination therapy and three patients as bridge-to-candidacy. The overall survival was 87.5 and 70% at 1 and 2 years, respectively. Freedom from stroke was 81.3% at 1 and 2 years. Freedom from device infection was 67.7 and 58.7% at 1 and 2 years, respectively. Freedom from gastrointestinal bleeding was 75 and 56.3% at 1 and 2 years, respectively. Freedom from readmission was 50 and 31.3% and at 6 months and 1 year, respectively.

**Conclusions:**

The Basel experience demonstrated the possible implementation of an LVAD program for destination therapy or bridge-to-candidacy in a non-transplant comprehensive heart-failure center with midterm survival results and freedom from major adverse events comparable to international registries. Patient selection remains crucial.

**Trial registration:**

This study was registered on the ClinicalTrials.gov database (NCT04263012).

## Background

Initially limited to patients with worsening end-stage heart failure awaiting heart transplant (HTx), left ventricular assist devices (LVADs) have undergone a tremendous development over the past three decades. Reduction in size and advancements in device safety have drastically improved patient survival and quality of life after LVAD implantation [1]. Due to the shortage of donor organs, bridge-to-recovery, bridge-to-decision, bridge-to-candidacy, and destination therapy (DT) have become commonplace practices [2]. In 2012, the American Heart Association recognized LVAD for DT in selected patients as a Class I recommendation, thereby impacting reimbursement policies in Switzerland [3]. Two years later, DT was granted with additional compensation nationally. Switzerland counts eight million inhabitants and five university hospitals, of which Zürich, Bern, and Lausanne. The latter offer HTx and long-term mechanical circulatory support. The University Hospital Basel (USB) is a 750-bed hospital with 7200 employees, is rated as a non-transplant, comprehensive heart-failure center (CHFC) and had performed HTx until 2005. From 2013, evidence of a growing heart transplant waiting list in our country prompted us to strengthen our heart failure treatment and extend our armamentarium [4]. As a non-transplant CHFC, we decided to prioritize patients aged 65 and above by implementing a mechanical-circulatory-support program for DT. The purpose of this study was to describe the initial set-up of an LVAD program, focusing on its interdisciplinary and comprehensive aspects, and share our 5-year clinical results.

## Methods

### Building up the core-team of the LVAD program

When planning an LVAD program, team-player selection is crucial. The team members must be willing to uphold a strong professional interdisciplinary experience. The LVAD core-team included heart-failure cardiologists, cardiac surgeons, cardiac anesthesiologists, intensivists, perfusionists, intensive care nurses, and ward nurses. We believe the most important position in an LVAD program to be the LVAD coordinator. This partner must be selected very carefully since LVAD coordination is the glue between all actors and disciplines. Moreover, the LVAD coordinator is in close contact with the patients and their relatives and coordinates the complex network of out-patient care. This network includes among others: general practitioners, dentists, rehabilitation-hospitals, home based nursing, and LVAD industry partners.

### Device selection, training, program setup and structure in brief

In 2013, while developing the program, we evaluated two different LVAD systems available on the market: HeartMate II (Abbott, Chicago, IL, USA), an axial continuous-flow pump and HeartWare HVAD (Medtronic, Minneapolis, MN, USA), a centrifugal continuous-flow pump. The latter presented the most modern propulsion concept. We opted for the HeartWare HVAD due to its recent promising clinical results, advanced hard- and software technology and its ease of implantation owing to smaller internal components [5]. Until October 2018, we implanted exclusively HeartWare HVAD. From 2019, influenced by the encouraging results from the MOMENTUM 3 trial, we switched to the next generation centrifugal pump HeartMate 3 (Abbott, Chicago, IL, USA) [6].

A group of six team members including an advanced heart failure cardiologist, cardiac surgeon, cardiac anesthesiologist, intensive care specialist, perfusionist, and the LVAD coordinator visited the Heart and Diabetes Center of Bad Oeynhausen (North Rhine-Westphalia, Germany). This center is one of the world’s largest in terms of mechanical circulatory support experience. Interviews of its LVAD program members, together with attendance of an LVAD implantation, made an important contribution to the architecture of our program. We further organized visitations of other international centers with extensive LVAD experience to gather a maximum amount of knowledge. In Basel, selected physicians and nurses from the cardiac surgery ward, the intensive care unit (ICU), the emergency department, and the operating theatre started an extensive in-house training.

### Patient selection, device implantation and postoperative follow-up

A multidisciplinary team evaluated patients with terminal heart failure and severely impaired exercise capacity despite optimal medical therapy as assessed by ergometry and 6-min walk test [7, 8]. The patients were not eligible for transplant because of either advanced age or non-modifiable comorbidities impacting their life expectancy (e.g., malignancy). We also considered younger candidates with bridge-to-candidacy profile, i.e. not eligible for transplant at the time of insertion due to modifiable comorbidities (e.g., morbid obesity). These latter candidates might eventually be addressed to a nearby transplantation center. We excluded patients with a life expectancy shorter than 12 months, younger than 18, with active infections, or with irreversible hepatic, renal, or neurological disorders. Further exclusion criteria were poor treatment compliance, psychiatric limitations, and poor social network. The preoperative check-up consisted of the following examinations: complete blood count and blood chemistry, computed angiography scan of the thorax, right- and left-heart catheterization, transthoracic echocardiography (including bubble study to exclude intra-cardiac shunts and left ventricular thrombi), carotid Doppler ultrasound, and dental exam. We evaluated the risk of postoperative right ventricular (RV) failure using predictive models [9, 10]. Patients with high probability of requiring RV assistance were not integrated in the elective destination therapy program. Patients with prior gastrointestinal bleeding had to undergo a gastroscopy and a colonoscopy. Additionally, the candidates had to consult a psychologist as well as a palliative care specialist to circumscribe their needs, desires, and expectations regarding the treatment. Finally, patients were included only upon acceptance from all core-team members.

The LVAD coordinator organized all necessary examinations and the implantation procedure itself. The LVAD coordinator was responsible for teaching the patients how to handle the device and its components (batteries, etc.), and how to deal with alarms. The patients ‘near-relatives as well as the hospital staff, rehabilitation staff and out-of-hospital caregivers were also instructed.

The program officially started in October 2014. The first implantation was performed with assistance of an external consultant cardiac surgeon with extensive experience in LVAD implantation. In the peri- and early postoperative phase, RV function was evaluated using both hemodynamic (such as central venous pressure, pulmonary pressures, and RV stroke work index) and echocardiographic parameters (such as RV size, contractility of the free wall, tricuspid annulus plane systolic excursion, tricuspid insufficiency, and position of the ventricular septum). A Swan-Ganz catheter was systematically inserted prior to surgery and left until hemodynamic stabilization. Transesophageal echocardiography was liberally performed until acute RV failure had been ruled-out and the patient had been weaned from inotropic drugs. In addition to adrenalin and intravenous milrinone, nebulized NO and/or iloprost were routinely administered to lower the pulmonary resistance and support the RV. After initial stabilization at the ICU, patients were transferred to the ward. There, we continued the educational program until patients could fully handle the device and batteries. We provided anticoagulation with intravenous heparin and subsequently with vitamin K antagonists in combination with Aspirin. To prevent cerebrovascular events, we targeted a mean arterial blood pressure below 85 mmHg [11]. We defined an internal protocol based on chlorhexidine disinfection and silver-coated cellulose for the driveline dressing. After discharge, all patients participated in a rehabilitation program in an institution specialized for heart-and-lung rehabilitation. After returning home, patients were monitored by the LVAD coordinator monthly. They consulted the cardiologist and the cardiac surgeon monthly during the first 6 months and at least quarter yearly subsequently. The cardiologist performed a transthoracic echocardiography to assess the right heart function and the competency of the valves. Based on clinical assessment, device readouts, and echocardiographic ramp tests if required, LVAD parameters were regularly adjusted to the patients’ needs.

We established a 24/7/365 directed phone chain and a standard-of-care algorithm for the out-patients to guarantee their permanent and direct hospital access. We designed a specific section of the ICU as well as of the cardiac surgery ward to house the LVAD patients, independently of their reason for admission. Furthermore, LVAD patients undergoing any non-cardiac surgical interventions had to be attended by a cardiac anesthesiologist as well as by a perfusionist.

The LVAD coordinator was responsible for planning the monthly LVAD meeting assembling all core-actors. During this meeting, we also evaluated new candidates with terminal heart failure referred by external general practitioners and cardiologists. Urgent patients were discussed in extraordinary meetings.

### Retrospective analysis

We reported common adverse events occurring after LVAD implantation as defined by the Interagency Registry for Mechanically Assisted Circulatory Support (INTERMACS) [12]. To visualize time-to-event outcomes, we used the Kaplan-Meier estimator. We defined as primary outcomes: overall survival, freedom from stroke excluding TIA and minor stroke (i.e., with non-disabling neurologic deficit), freedom from device infection including driveline exit site infection, freedom from gastro-intestinal bleeding (GIB), and freedom from readmission due to LVAD- or cardiac related complications. We defined as first major adverse event after LVAD implantation the first occurrence of one the following: device infection, clinically significant bleeding (defined as requiring hospitalization for transfusion, endoscopy, or surgical intervention), device malfunction (defined as pump thrombosis or any dysfunction requiring LVAD explantation), stroke, or death. To investigate the evolution of dyspnea 3 months after implantation, we used a symmetry test on repeated NYHA assessments.

We described continuous variables as mean ± standard error or median (range minimum – maximum), categorical variables as number with percentage, survivals as percentage with 95% confidence intervals (CI). We carried out the statistical analysis using SPSS Statistics 24 (IBM, Armonk, NY, USA) and Stata 15 (Stata Corp., College Station, TX, USA). We compared our results to internationally reported data. This study was registered on the ClinicalTrials.gov database (NCT04263012).

## Results

### Perioperative patient characteristics

Between October 2014 and September 2019, 16 patients received an LVAD in our center. Most of the patients were male with a mean age at implantation of 67.1 years ±2.6. The mean preoperative EuroSCORE II was 24.4% ± 3.9 and the median INTERMACS level was 4 (range 1–5). Thirteen patients received an LVAD as DT and three patients as bridge-to-candidacy. The most frequent etiology of heart failure was ischemic, the remaining consisting of idiopathic dilated and valvular cardiomyopathies. Patients with prior cardiac surgery accounted for 37.5%. A concomitant operative procedure was performed in 75% of the patients including tricuspid valve repair, coronary artery bypass graft, mitral valve repair, and others. We implanted all LVAD in an on-pump, beating heart fashion with a mean cardiopulmonary bypass time of 139 min ± 10. Tricuspid valve repair was also performed beating heart. Aortic cross-clamp was limited to additional procedures on the left heart. Thirteen patients received HeartWare HVAD and three patients received HeartMate 3. The clinical follow-up ended on December 31, 2019. Table 1 details the patients ’demographics and perioperative characteristics.

**Table 1:** Patients’ demographics and perioperative characteristics

Variable	n (%) or mean ***±*** SE
Age	67.1 ± 2.6
Gender	
male	14 (87.5)
female	2 (12.5)
BMI (kg/m^2^)	26.3 ± 1
Diabetes mellitus on insulin	1 (6.2)
COPD	7 (43.8)
GFR < 45 mL/min/1.73 m^2^	13 (81.3)
Nicotine (active)	2 (12.5)
Atrial fibrillation	10 (62.5)
Peripheral vascular disease	2 (12.5)
ICD	14 (87.5)
CRT	12 (75)
Cardiomyopathy	
ischemic	12 (75)
idiopathic dilated	3 (18.8)
valvular	1 (6.2)
NYHA class	
III	10 (62.5)
IV	6 (37.5)
INTERMACS profile	
1	1 (6.2)
3	1 (6.2)
4	7 (43.8)
5	7 (43.8)
Intention	
DT	13 (81.3)
BTC	3 (18.7
Concomitant procedure	
TV repair	1 (6.2)
CABG	2 (12.5)
MV repair	2 (12.5)
AV repair	1 (6.2)
AVR (bioprosthesis)	1 (6.2)
PFO closure	1 (6.2)
Prior cardiac surgery	6 (37.5)
Device implanted	
HeartWare HVAD	13 (81.3)
HeartMate 3	3 (18.7)
EuroSCORE II (%)	24.4 ± 3.9

*SE* standard error, *BMI* body mass index, *COPD* chronic obstructive pulmonary disease, *GFR* glomerular filtration rate, *ICD* implantable cardiac defibrillator, *CRT* cardiac resynchronization therapy, *NYHA* New York Heart Association, *INTERMACS* Interagency Registry for Mechanically Assisted Circulatory Support, *DT* destination therapy, *BTC* bridge-to-candidacy, *TV* tricuspid valve, *CABG* coronary artery bypass graft, *MV* mitral valve, *AV* aortic valve, *AVR* aortic valve replacement, *PFO* permanent foramen ovale

### Overall survival and freedom from major adverse events

All patients survived the operation as well as their hospital course and rehabilitation stay. We observed a single case of severe right heart failure during the immediate postoperative course. Short-term right ventricle support with provisory implantation of an Impella RP® (ABIOMED Inc., Danvers, MA, USA) successfully restored the right ventricle function. We discharged the patients from the ICU after a median stay of 9.5 days (range 3–41). Five patients required early surgical re-exploration due to bleeding. We observed mostly diffuse bleeding. No surgical revision of the anastomosis or of the core-site was required.

**Fig. 1:**
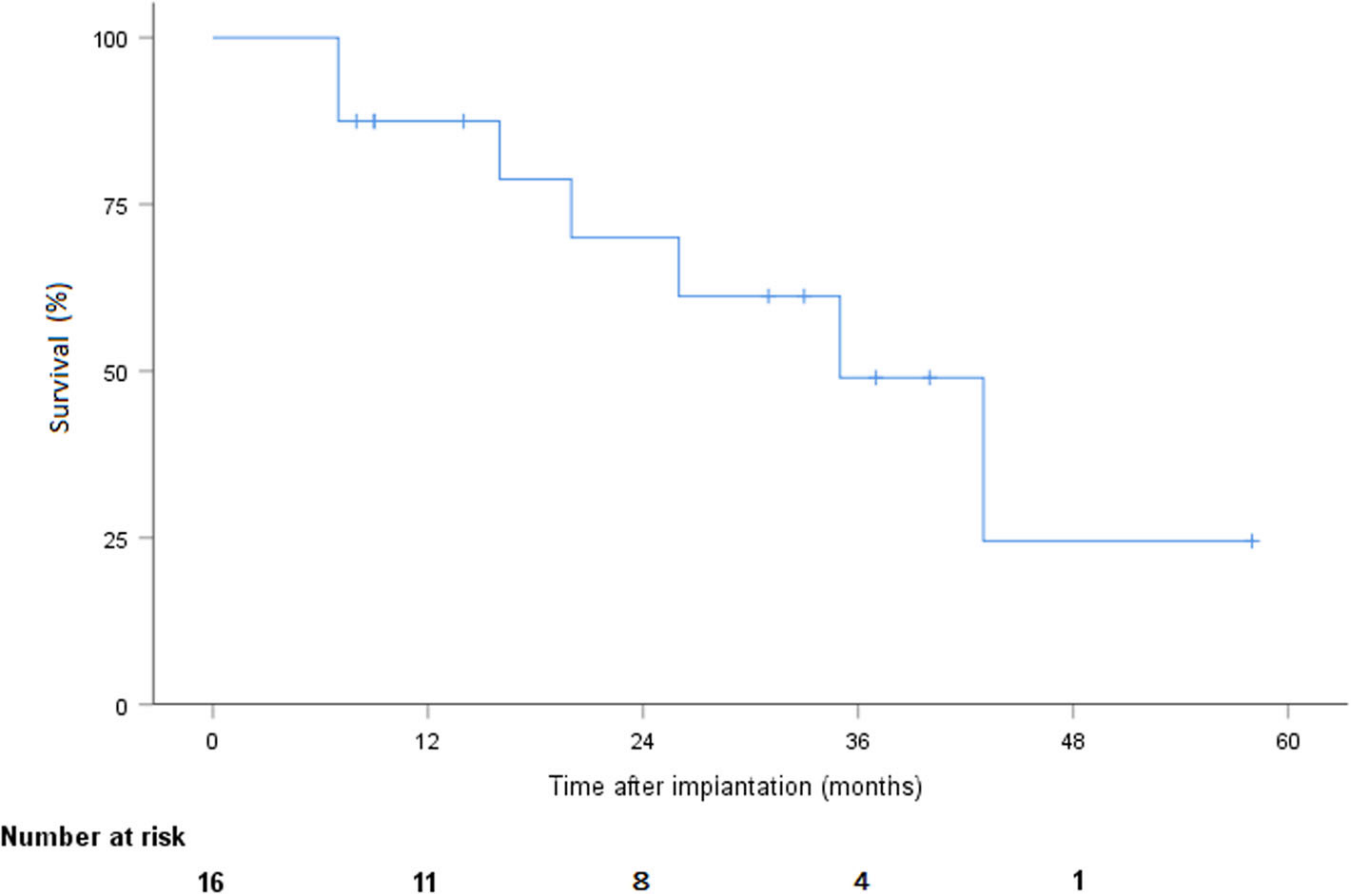
Overall survival after LVAD implantation

**Fig. 2:**
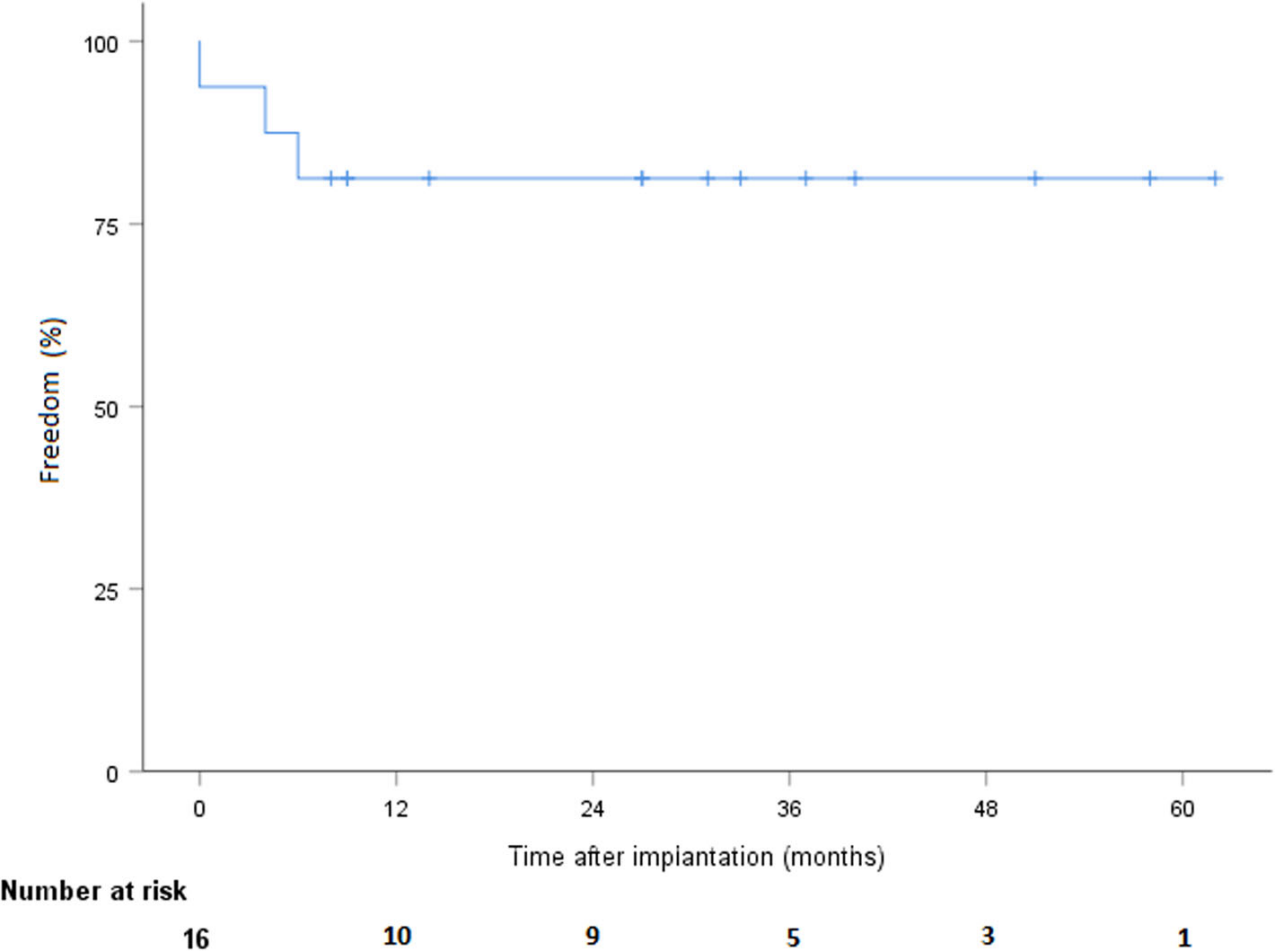
Freedom from stroke after LVAD implantation

**Fig. 3:**
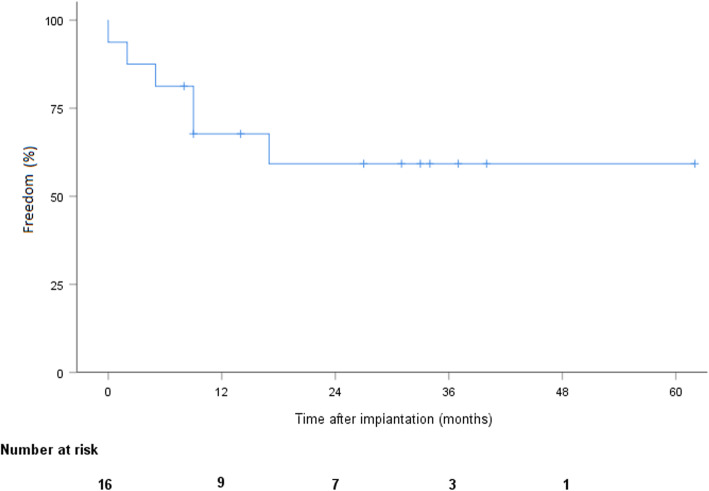
Freedom from major infection after LVAD implantation

**Fig. 4:**
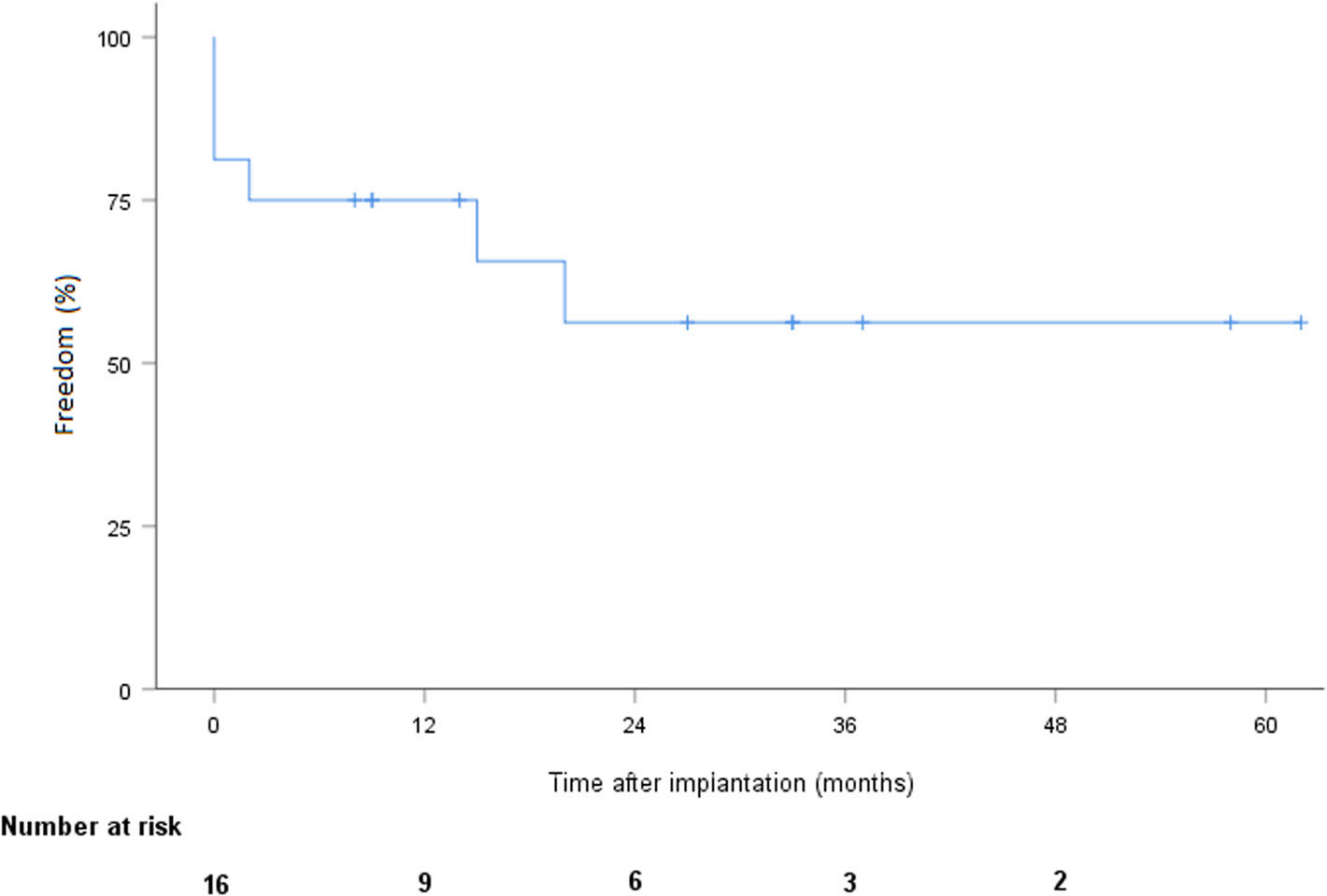
Freedom from gastrointestinal bleeding after LVAD implantation

During follow-up, 7 patients died after a mean time of 22 months ±5.2, including 5 from cardiac or LVAD-related conditions: multiple organ failure (*n* = 1), renal failure (*n* = 1), hemorrhagic shock secondary to systemic infection (*n* = 1), and neurological complications (*n* = 2). Among the deceased patients, 5 had been implanted as DT and 2 as bridge-to-candidacy. According to the Kaplan-Meier estimate, overall survival was 87.5% (CI 58.6–96.7), 70% (CI 37.5–87.8), and 49% (CI 18–74.3) at 1, 2, and 3 years, respectively (Fig. 1). Pump thrombosis occurred in 6 patients after a mean time of 16.8 months ±6. Pump thrombosis was diagnosed based on elevated LDH levels (at least twice the upper limit of normal) and sudden, sustained pump power elevation (> 1 W). All were treated successfully with systemic lysis therapy without further complication. No intracranial hemorrhage resulted from thombolytics. One patient required multiple lysis over several months due to recurrent / persistent thrombosis. The adjunction of clopidogrel to aspirin and vitamine K antagonist was finally successful in treating the thrombosis. No pump explantation was required due to thrombosis. We did not observe any other dysfunction of both devices. Stroke occurred in 3 patients, including hemorrhagic and ischemic insults. According to the Kaplan-Meier estimate, freedom from stroke was 81.3% (CI 52.5–93.5) at 1 and 2 years (Fig. 2). Furthermore, 5 patients experienced transient ischemic attacks or minor strokes with complete regression of symptoms within a few hours in all cases. Device infection occurred in 6 patients, including infections limited to the driveline exit site (*n* = 3) and systemic infection (*n* = 3) requiring long-term antibiotic therapy. Two cases of driveline infection required surgical debridement. According to the Kaplan-Meier estimate, freedom from device infection was 67.7% (CI 37.7–84.9) and 58.7% (CI 29.4–79.2) at 1 and 2 years, respectively (Fig. 3). No pump explantation was required due to infection. GIB occurred in 6 patients. According to Kaplan-Meier estimate, freedom from GIB was 75% (CI 46.3–89.8) and 56.3% (CI 26.0–78.2) at 1 and 2 years, respectively (Fig. 4). After discharge, all patients were eventually readmitted due to cardiac- or LVAD-related complications. The mean time to readmission was 8.6 months ±2.1. According to the Kaplan-Meier estimate, freedom from readmission was 50% (CI 25.5–74.5) and 31.3% (CI 8.6–54) and at 6 months and 1 year, respectively. Table 2 summarizes the postoperative follow-up and adverse events.

**Table 2:** Postoperative follow-up and adverse events after LVAD implantation

Variable	n (%)
Reoperation for bleeding	5 (31.3)
Right heart failure requiring circulatory assistance	1 (6.2)
Device infection	
driveline exit site	3 (18.8)
systemic	3 (18.8)
Pump thrombosis	6 (37.5)
Stroke	
ischemic	2 (12.5)
hemorrhagic	1 (6.2)
Gastro-intestinal bleeding	6 (37.5)
NYHA class after 3 months	
II	6 (37.5)
III	3 (18.8)
Readmission for cardiac or LVAD-related complications	16 (100)
Death	
cardiac / LVAD-related	5 (31.3)
non-cardiac	2 (12.5)

*LVAD* left-ventricular assist device

### Freedom from first major adverse event

Freedom from a first major adverse event was estimated at 50% (CI 24.5–71.1) and 43.8% (CI 19.8–65.6) at 6 months and 1 year, respectively.

### Evolution of dyspnea

Based on NYHA assessment, dyspnea after 3 months was statistically significantly reduced according to the symmetry test (*p* = 0.04).

### Comparison to internationally reported data

We compared the previous findings to international LVAD registries based on the following reports: the eighth annual INTERMACS, the European Registry for Patients with Mechanical Circulatory Support (EUROMACS) 2018, and the Society of Thoracic Surgeons (STS) 2019 [13–15]. Table 3 summarizes the comparison of our data to those internationally reported.

**Table 3:** Comparison of internationally reported overall survival and freedom from major adverse events after LVAD-Implantation

Outcome	USB		INTERMACS 2017^a^		EUROMACS 2018		STS 2019^b^	
	1 year	2 years	1 year	2 years	1 year	2 years	1 year	2 years
Overall survival	87.5%	70%	84%	74%	69%	55%	81%	72%
Freedom from stroke	81.3%	81.3%	86%	82%	*NR*	*NR*	83%	80%
Freedom from device infection	67.7%	58.7%	86%	80%	*NR*	*NR*	60%	49%
Freedom from gastrointestinal bleeding	75%	56.3%	*NR*	*NR*	*NR*	*NR*	80%	75%

*LVAD* left-ventricular assist device, *USB* University Hospital of Basel, *INTERMACS* Interagency Registry for Mechanically Assisted Circulatory Support, *EUROMCAS* European Registry for Patients with Mechanical Circulatory Support, *STS* Society of Thoracic Surgeons, *NR* not reported

^a^patients with specifically high INTERMACS profiles (4–7)

^b^patients after implantation of continuous-flow hybrid levitation devices

Comparing the INTERMACS cohort with specifically high INTERMACS scores (4–7), we reported a similar short-term overall survival (87.5% versus 84 and 70% versus 74% at 1 and 2 years, respectively). In terms of morbidity, we reported a lower short-term freedom from stroke (81.3% versus 86 and 81.3% versus 82% at 1 and 2 years, respectively), lower freedom from device infection (67.7% versus 86 and 58.7% versus 80% after 1 and 2 years, respectively), higher freedom from readmission (50% versus 39% after 6 months), and higher freedom from first major event (50% versus 45 and 43.8% versus 32% at 6 months and 1 year, respectively).

Compared to the EUROMACS cohort, we reported a higher overall survival (87.5% versus 69 and 70% versus 55% at 1 and 2 years, respectively).

Comparing the specific subgroup implanted with centrifugal-flow hybrid levitation devices (i.e. HVAD) of the STS cohort, we reported a higher short-term overall survival (87.5% versus 79 and 70% versus 69% at 1 and 2 years, respectively). We reported a comparable freedom from stroke (81.3 versus 84% und 81.3% versus 78% at 1 and 2 years, respectively), higher freedom from device infection (67.7% versus 57 and 58.7% versus 45% at 1 and 2 years, respectively), and lower freedom from GIB (freedom from GIB of 75% versus 80 and 56.3% versus 74% after 1 and 2 years, respectively).

## Discussion

The increasing prevalence of advanced heart failure combined with population aging in western countries and limited availability of donor organs, account for the growing interest for LVAD as DT in the recent years. By depicting the set-up of our program, we sought to provide practical elements for similar-sized centers wishing to implement a mechanical-circulatory-support program for DT. To our knowledge, only a few center’s experience of establishing an LVAD-program are available with long-term results in the literature, and none specifically for DT [16–21]. Those available with long-term results usually concerned older-generation devices with different implantation strategies from those in our study (i.e. mainly bridge-to-transplant). Therefore, and to more objectively assess the outcomes of our cohort, we instead compared them to international LVAD registries, selecting, where appropriate, the subgroups whose profile better matched our cohort.

### Overall survival

When comparing the INTERMACS cohort with specifically high INTERMACS profile patients (4–7), we observed an equivalent overall short-term survival. In the longer term, our patients’ survival was slightly lower. Nonetheless, this finding must be nuanced by the composition of the INTERMACS cohort, which includes patients with both strategies of DT and bridge-to-transplant (7389 versus 4761 for DT and bridge-to-transplant, respectively for the whole INTERMACS cohort). For DT patients are known for higher mortality, we may have expected a lower survival in our patients.

We interpreted the higher overall survival compared to EUROMACS due to the disparity of INTERMACS profiles between the cohorts (12.5% versus 69% of INTERMACS < 4 in the USB and EUROMACS cohort, respectively).

The STS 2019 report details the clinical outcomes of 3 different cohorts defined by the type of implanted devices: axial flow, centrifugal-flow with hybrid levitation, and centrifugal-flow with fully magnetic levitation. HeartWare HVAD corresponds to the second category. As our patients were mostly implanted with HeartWare HVAD, we chose to refer to this cohort. As with EUROMACS, the higher short-term survival of our patients was explainable by the disparity of INTERMACS profiles between the cohorts (12.5% versus 89.1% of INTERMACS < 4 in the USB and STS cohort, respectively).

### Major adverse events

All strokes occurred during the first 6 months, explaining the relatively high short-term incidence of strokes. In the longer term, freedom from strokes was similar to that internationally reported. The overall high rate of neurologic events in this study may be explained by the device initially chosen, as HeartWare HVAD has been associated with higher rates of strokes compared both to HeartMate II and HeartMate 3 [22, 23]. The latter were predominantly implemented in the three mentioned international LVAD registries.

We observed a comparable freedom from infection to that of the STS registry but a notably lower to that of the INTERMACS registry. The literature mentions up to 51.9% infections after implantation of continuous-flow LVADs [24]. A recent review mentions 19–39% infection rates after implantation of second- and third-generation LVADs [25]. A Swiss group recently reported 45% LVAD-related infection after HeartMate 3 implantation, including driveline ex-site infections [26]. The latter rates correspond to our results (37.6%). 50% of the device-related infections in our cohort were limited to the driveline ex-site. Currently, risk factors for LVAD post-implantation infections have not been clearly identified. However, DT has been associated with increased susceptibility for device infection [24].

We reported a high occurrence of GIB. GIB is a common complication occurring in up to 61% of patients after LVAD implantation, presumably due to the combination of angiodysplasia formation and acquired von Willebrand syndrome resulting from the continuous-flow mechanical circulation [27]. Although controversial, evidence suggests that the risk of bleeding associated with angiodysplasia after LVAD implantation increases with age [28]. Angiodysplasia is sometimes a difficult diagnose that can be missed by endoscopy [29]. Out of 6 cases of GI-Bleeding in our cohort, 1 was due to angiodysplasia, 1 to gastric ulcer, 1 to colon ulcer, 1 to radiation proctitis, and 2 remained of unknown origin despite comprehensive work-up including upper, lower, and capsule endoscopy. All patients with GIB were implanted as DT with a mean age of 69.7 years. This finding suggests that the advanced age of our patients may have contributed to the high incidence of GIB, presumably due to undiagnosed angiodysplasia. The introduction of octreotide drastically reduced the recurrence of bleeding in our patients with diagnosed angiodysplasia or for whom the bleeding site could not be identified with endoscopy.

### Study limitations

This study was limited by the small number of patients. Finding comparable data in the literature to critically assess the outcomes of our cohort was difficult. Indeed, the cohort was composed of highly selected patients (median INTERMACS score 4), mainly implanted as DT, and mainly with a single device (HeartWare HVAD). The number of patients was obviously too small to be statistically compared to cohorts such as those of INTERMACS, STS or EUROMACS. Thus, we mentioned the results of these registries as a reference and estimated the “trend” (higher, equivalent, or lower) between our results and those from the reports mentioned. We compared survivals from the INTERMACS registry specific for patients with high INTERMACS profile, since these better matched our cohort. For the same reason, we compared survivals from the STS registry specific for patients after implantation of centrifugal-flow hybrid levitation devices (i.e. HeartWare HVAD).

Our cohort consisted mostly of “frequent flyers” (INTERMACS profile 4), for we did not intend to primarily implant LVADs in inotrope-dependent, instable patients while starting the program. However, the high EuroSCORE II (24.4%) underlined the high morbidity of the included patients.

Finally, we did not specifically attest the patients’ quality of life of with a questionnaire. Nevertheless, the statistically significant reduction of dyspnea 3 months after LVAD implantation highlighted the subjective improvement of the patients’ condition.

## Conclusions

The Basel experience demonstrated the possible implementation of an LVAD program for DT or bridge-to-candidacy in a non-transplant CHFC with midterm survival results and freedom from major adverse events comparable to international registries. This study was designed to detail practical, useful aspects for centers wishing to develop a mechanical-circulatory-support program for DT. Our findings are encouraging for similar-sized centers, for they somewhat contradicted previously reported data suggesting that low center volume negatively impacts clinical outcomes after LVAD implantation [30]. Patient selection remains crucial.

## Data Availability

The datasets used and/or analyzed during the current study are available from the corresponding author on reasonable request.

## Abbreviations

HTx: Heart transplant
LVAD: Left-ventricular assist device
DT: Destination therapy
USB: University Hospital of Basel
CHFC: Comprehensive heart-failure center
ICU: Intensive care unit
RV: Right ventricle
INTERMACS: Interagency Registry for Mechanically Assisted Circulatory Support
GIB: Gastrointestinal bleeding
